# Supplementary material for: The Relationship Between Body, Mind, and Social Characteristics in a Sample of a Training Program for Developing Social and Personal Resources: A Network Analysis
Source: Int J Environ Res Public Health. 2024 Dec 11;21(12):1654. doi: 10.3390/ijerph21121654 (PMC11675297; doi:10.3390/ijerph21121654)
Supplement: Supplementary file 1 [file ijerph-21-01654-s001.zip › ijerph-3321824-supplementary.pdf]

Table S1: Weights Matrix for the pairwise interactions of variables in the Unified Network

	Network																							
**Variable**	**1**	**2**	**3**	**4**	**5**	**6**	**7**	**8**	**9**	**10**	**11**	**12**	**13**	**14**	**15**	**16**	**17**	**18**	**19**	**20**	**21**	**22**	**23**	**24**
**1**	0.000	-0.114	-0.132	-0.214	0.000	0.000	-0.133	0.000	0.000	-0.154	-0.085	0.000	0.000	0.000	0.086	0.000	0.000	-0.081	0.000	-0.064	-0.061	0.000	0.000	0.000
**2**	-0.114	0.000	0.000	0.150	0.000	0.000	0.064	0.000	0.000	0.198	0.061	0.000	0.000	0.000	0.000	0.000	0.000	0.000	0.000	0.000	0.000	0.000	0.000	0.000
**3**	-0.132	0.000	0.000	0.130	0.000	0.000	0.151	0.000	0.138	0.000	0.000	0.206	0.000	0.137	0.000	0.000	0.000	0.000	0.000	0.000	0.000	0.000	0.000	0.000
**4**	-0.214	0.150	0.130	0.000	0.000	0.302	0.204	0.000	0.000	0.000	0.000	0.000	0.000	0.000	0.000	0.000	0.000	0.000	0.000	0.000	0.000	0.000	0.000	0.000
**5**	0.000	0.000	0.000	0.000	0.000	0.176	0.000	0.000	0.000	0.000	0.000	0.000	0.000	0.000	0.000	0.000	0.080	0.000	0.000	0.000	0.000	0.000	0.000	0.000
**6**	0.000	0.000	0.000	0.302	0.176	0.000	0.000	0.000	0.000	0.178	0.000	0.000	0.000	0.000	0.000	0.000	0.000	0.000	0.000	0.000	0.000	0.000	0.000	0.000
**7**	-0.133	0.064	0.151	0.204	0.000	0.000	0.000	0.128	0.000	0.132	0.000	0.000	0.000	0.000	0.000	0.000	0.210	0.000	0.000	0.069	0.000	0.000	0.000	0.000
**8**	0.000	0.000	0.000	0.000	0.000	0.000	0.128	0.000	0.000	0.000	0.000	0.125	0.000	0.185	0.000	0.000	0.000	-0.163	0.000	0.079	0.000	0.000	0.129	0.000
**9**	0.000	0.000	0.138	0.000	0.000	0.000	0.000	0.000	0.000	0.054	0.000	0.000	0.000	0.101	0.184	0.000	0.106	0.000	0.095	0.079	0.000	0.125	0.043	0.270
**10**	-0.154	0.198	0.000	0.000	0.000	0.178	0.132	0.000	0.054	0.000	0.117	0.000	0.080	0.000	0.000	0.000	0.261	0.000	0.000	0.000	0.000	0.000	0.000	0.000
**11**	-0.085	0.061	0.000	0.000	0.000	0.000	0.000	0.000	0.000	0.117	0.000	0.000	0.000	0.164	0.225	0.146	0.000	0.000	0.000	0.000	0.039	0.000	0.000	0.000
**12**	0.000	0.000	0.206	0.000	0.000	0.000	0.000	0.125	0.000	0.000	0.000	0.000	0.000	0.306	0.000	0.000	0.000	0.000	-0.060	0.000	0.000	0.000	0.000	0.137
**13**	0.000	0.000	0.000	0.000	0.000	0.000	0.000	0.000	0.000	0.080	0.000	0.000	0.000	0.107	0.256	0.082	0.240	0.000	0.000	-0.171	0.000	0.000	-0.133	0.200
**14**	0.000	0.000	0.137	0.000	0.000	0.000	0.000	0.185	0.101	0.000	0.164	0.306	0.107	0.000	-0.302	-0.169	0.089	0.000	-0.074	0.000	-0.067	0.000	0.000	0.151
**15**	0.086	0.000	0.000	0.000	0.000	0.000	0.000	0.000	0.184	0.000	0.225	0.000	0.256	-0.302	0.000	-0.115	0.087	0.000	-0.114	0.000	0.000	0.127	0.000	0.309
**16**	0.000	0.000	0.000	0.000	0.000	0.000	0.000	0.000	0.000	0.000	0.146	0.000	0.082	-0.169	-0.115	0.000	0.000	0.000	0.269	0.000	-0.075	0.000	0.000	0.285
**17**	0.000	0.000	0.000	0.000	0.080	0.000	0.210	0.000	0.106	0.261	0.000	0.000	0.240	0.089	0.087	0.000	0.000	0.000	0.000	0.000	0.000	0.000	0.218	0.000
**18**	-0.081	0.000	0.000	0.000	0.000	0.000	0.000	-0.163	0.000	0.000	0.000	0.000	0.000	0.000	0.000	0.000	0.000	0.000	0.000	0.265	0.000	0.000	0.318	0.400
**19**	0.000	0.000	0.000	0.000	0.000	0.000	0.000	0.000	0.095	0.000	0.000	-0.060	0.000	-0.074	-0.114	0.269	0.000	0.000	0.000	0.000	0.000	0.000	0.000	-0.233
**20**	-0.064	0.000	0.000	0.000	0.000	0.000	0.069	0.079	0.079	0.000	0.000	0.000	-0.171	0.000	0.000	0.000	0.000	0.265	0.000	0.000	0.061	-0.119	0.000	0.101
**21**	-0.061	0.000	0.000	0.000	0.000	0.000	0.000	0.000	0.000	0.000	0.039	0.000	0.000	-0.067	0.000	-0.075	0.000	0.000	0.000	0.061	0.000	-0.036	0.000	0.183
**22**	0.000	0.000	0.000	0.000	0.000	0.000	0.000	0.000	0.125	0.000	0.000	0.000	0.000	0.000	0.127	0.000	0.000	0.000	0.000	-0.119	-0.036	0.000	0.000	0.000
**23**	0.000	0.000	0.000	0.000	0.000	0.000	0.000	0.129	0.043	0.000	0.000	0.000	-0.133	0.000	0.000	0.000	0.218	0.318	0.000	0.000	0.000	0.000	0.000	0.000
**24**	0.000	0.000	0.000	0.000	0.000	0.000	0.000	0.000	0.270	0.000	0.000	0.137	0.200	0.151	0.309	0.285	0.000	0.400	-0.233	0.101	0.183	0.000	0.000	0.000

Table S2: Centrality Indices and expected influence measures of variables in the Unified Network

	Network		
**Variable**	**Betweenness**	**Closeness**	**Strength**
**1**	-0.539	-0.373	0.184
**2**	-0.804	-1.385	-0.984
**3**	0.097	-0.051	-0.316
**4**	0.150	-0.732	-0.087
**5**	-0.804	-2.145	-1.705
**6**	-0.274	-1.093	-0.835
**7**	-0.168	-0.003	0.111
**8**	-0.751	-0.086	-0.500
**9**	-0.327	0.608	0.343
**10**	0.892	0.110	0.294
**11**	-0.486	0.253	-0.439
**12**	-0.804	0.094	-0.449
**13**	1.211	1.345	0.502
**14**	0.150	0.565	1.773
**15**	0.945	1.591	1.669
**16**	-0.698	-0.018	0.221
**17**	2.112	1.137	0.554
**18**	0.733	1.304	0.408
**19**	-0.804	-0.619	-0.424
**20**	-0.698	-0.199	-0.069
**21**	-0.804	-1.033	-1.129
**22**	-0.804	-1.494	-1.374
**23**	-0.433	0.726	-0.430
**24**	2.907	1.498	2.681

Table S3: Weights Matrix for the pairwise interactions of variables in the Control Group Network

	Network																						
**Variable**	**1**	**2**	**3**	**4**	**5**	**6**	**7**	**8**	**9**	**10**	**11**	**12**	**13**	**14**	**15**	**16**	**17**	**18**	**19**	**20**	**21**	**22**	**23**
**1**	0,000	-0,076	-0,146	-0,177	0,000	0,000	-0,172	0,000	0,000	-0,197	0,000	0,000	0,000	0,000	0,103	0,000	0,000	0,000	0,000	0,000	-0,062	0,000	0,000
**2**	-0,076	0,000	0,000	0,208	0,000	0,000	0,000	0,000	0,000	0,211	0,000	0,000	0,000	0,104	0,000	0,000	0,000	0,000	0,000	0,000	0,000	0,000	0,000
**3**	-0,146	0,000	0,000	0,147	0,000	0,000	0,207	0,000	0,102	0,000	0,000	0,129	0,000	0,152	0,000	0,000	0,000	0,101	0,000	0,000	0,000	0,000	0,000
**4**	-0,177	0,208	0,147	0,000	0,000	0,264	0,160	0,000	0,000	0,000	0,000	0,000	0,000	0,000	0,000	0,000	0,000	0,000	0,000	0,000	0,000	0,000	0,000
**5**	0,000	0,000	0,000	0,000	0,000	0,208	0,000	0,000	0,000	0,000	0,000	0,000	0,000	0,092	0,000	0,000	0,000	0,000	0,000	0,000	0,000	0,000	0,000
**6**	0,000	0,000	0,000	0,264	0,208	0,000	0,000	0,000	0,000	0,227	0,000	0,000	0,000	0,000	0,000	0,000	0,000	0,000	0,000	0,000	0,000	0,000	0,000
**7**	-0,172	0,000	0,207	0,160	0,000	0,000	0,000	0,072	0,000	0,129	0,000	0,000	0,000	0,000	0,000	0,000	0,154	0,000	0,000	0,000	0,000	0,000	0,000
**8**	0,000	0,000	0,000	0,000	0,000	0,000	0,072	0,000	0,000	0,000	0,000	0,145	0,000	0,163	0,000	0,000	0,000	0,000	0,000	0,000	0,000	0,000	0,000
**9**	0,000	0,000	0,102	0,000	0,000	0,000	0,000	0,000	0,000	0,000	0,000	0,000	0,000	0,000	0,096	0,093	0,000	0,000	0,000	0,000	0,000	0,000	0,035
**10**	-0,197	0,211	0,000	0,000	0,000	0,227	0,129	0,000	0,000	0,000	0,000	0,000	0,091	0,000	0,000	0,000	0,293	0,000	0,000	0,000	0,000	0,000	0,000
**11**	0,000	0,000	0,000	0,000	0,000	0,000	0,000	0,000	0,000	0,000	0,000	0,000	0,000	0,144	0,223	0,212	0,000	0,000	0,000	0,000	0,071	0,000	0,000
**12**	0,000	0,000	0,129	0,000	0,000	0,000	0,000	0,145	0,000	0,000	0,000	0,000	0,000	0,320	0,000	0,000	0,000	0,000	0,000	0,000	0,000	0,000	0,000
**13**	0,000	0,000	0,000	0,000	0,000	0,000	0,000	0,000	0,000	0,091	0,000	0,000	0,000	0,000	0,251	0,000	0,132	0,000	0,000	-0,150	0,000	0,000	0,000
**14**	0,000	0,104	0,152	0,000	0,092	0,000	0,000	0,163	0,000	0,000	0,144	0,320	0,000	0,000	-0,252	-0,246	0,187	0,000	0,000	0,000	-0,061	0,000	0,000
**15**	0,103	0,000	0,000	0,000	0,000	0,000	0,000	0,000	0,096	0,000	0,223	0,000	0,251	-0,252	0,000	-0,141	0,124	0,000	0,000	0,000	0,000	0,000	0,000
**16**	0,000	0,000	0,000	0,000	0,000	0,000	0,000	0,000	0,093	0,000	0,212	0,000	0,000	-0,246	-0,141	0,000	0,000	0,000	0,347	0,000	-0,106	0,000	0,000
**17**	0,000	0,000	0,000	0,000	0,000	0,000	0,154	0,000	0,000	0,293	0,000	0,000	0,132	0,187	0,124	0,000	0,000	0,000	-0,087	0,000	0,000	0,000	0,252
**18**	0,000	0,000	0,101	0,000	0,000	0,000	0,000	0,000	0,000	0,000	0,000	0,000	0,000	0,000	0,000	0,000	0,000	0,000	0,000	0,267	0,000	0,000	0,358
**19**	0,000	0,000	0,000	0,000	0,000	0,000	0,000	0,000	0,000	0,000	0,000	0,000	0,000	0,000	0,000	0,347	-0,087	0,000	0,000	0,000	0,000	0,000	0,000
**20**	0,000	0,000	0,000	0,000	0,000	0,000	0,000	0,000	0,000	0,000	0,000	0,000	-0,150	0,000	0,000	0,000	0,000	0,267	0,000	0,000	0,094	-0,096	0,000
**21**	-0,062	0,000	0,000	0,000	0,000	0,000	0,000	0,000	0,000	0,000	0,071	0,000	0,000	-0,061	0,000	-0,106	0,000	0,000	0,000	0,094	0,000	-0,041	0,000
**22**	0,000	0,000	0,000	0,000	0,000	0,000	0,000	0,000	0,000	0,000	0,000	0,000	0,000	0,000	0,000	0,000	0,000	0,000	0,000	-0,096	-0,041	0,000	0,000
**23**	0,000	0,000	0,000	0,000	0,000	0,000	0,000	0,000	0,035	0,000	0,000	0,000	0,000	0,000	0,000	0,000	0,252	0,358	0,000	0,000	0,000	0,000	0,000

Table S4: Weights Matrix for the pairwise interactions of variables in the Training Group Network

	Network																						
**Variable**	**1**	**2**	**3**	**4**	**5**	**6**	**7**	**8**	**9**	**10**	**11**	**12**	**13**	**14**	**15**	**16**	**17**	**18**	**19**	**20**	**21**	**22**	**23**
**1**	0,000	-0,119	0,000	-0,261	0,000	-0,165	0,000	0,000	0,000	-0,092	-0,112	0,000	0,000	0,000	0,000	0,000	0,000	0,000	0,000	0,000	0,000	0,000	0,000
**2**	-0,119	0,000	0,192	0,000	0,000	0,000	0,000	0,000	0,000	0,000	0,124	0,000	0,000	0,000	0,000	0,000	0,000	0,000	0,000	0,000	0,000	0,000	0,000
**3**	0,000	0,192	0,000	0,000	0,000	0,000	0,000	0,000	0,283	0,275	0,000	0,319	0,000	0,000	0,000	0,000	0,000	0,000	0,000	0,000	0,000	0,000	0,000
**4**	-0,261	0,000	0,000	0,000	0,000	0,273	0,274	0,000	0,000	0,000	0,000	0,000	0,000	0,000	0,000	0,000	0,000	0,000	0,000	0,000	0,000	0,000	0,000
**5**	0,000	0,000	0,000	0,000	0,000	0,000	0,000	0,000	0,000	0,000	0,000	0,000	0,000	0,000	0,000	0,000	0,000	0,000	0,000	0,000	0,000	0,000	0,000
**6**	-0,165	0,000	0,000	0,273	0,000	0,000	0,000	0,000	0,000	0,000	0,000	0,000	0,000	0,000	0,000	0,000	0,000	0,000	0,000	0,000	0,000	0,000	0,000
**7**	0,000	0,000	0,000	0,274	0,000	0,000	0,000	0,162	0,083	0,105	0,000	0,000	0,000	0,000	0,000	0,000	0,335	0,000	0,000	0,000	0,000	0,000	0,000
**8**	0,000	0,000	0,000	0,000	0,000	0,000	0,162	0,000	0,000	0,000	0,000	0,000	0,000	0,000	0,000	0,098	0,000	0,000	0,000	0,000	0,000	0,000	0,000
**9**	0,000	0,000	0,283	0,000	0,000	0,000	0,083	0,000	0,000	0,000	0,000	0,000	0,000	0,000	0,000	0,000	0,169	0,122	0,233	0,000	0,000	0,000	0,000
**10**	-0,092	0,000	0,275	0,000	0,000	0,000	0,105	0,000	0,000	0,000	0,117	0,000	0,000	0,000	0,000	0,000	0,164	0,000	0,000	0,000	0,000	0,000	0,000
**11**	-0,112	0,124	0,000	0,000	0,000	0,000	0,000	0,000	0,000	0,117	0,000	0,000	0,000	0,210	0,194	0,000	0,000	-0,137	0,000	0,000	0,000	0,000	0,000
**12**	0,000	0,000	0,319	0,000	0,000	0,000	0,000	0,000	0,000	0,000	0,000	0,000	0,000	0,120	0,000	0,000	0,000	0,000	0,000	0,000	0,000	0,000	0,000
**13**	0,000	0,000	0,000	0,000	0,000	0,000	0,000	0,000	0,000	0,000	0,000	0,000	0,000	0,000	0,000	0,000	0,311	0,000	0,000	0,000	0,000	0,000	-0,193
**14**	0,000	0,000	0,000	0,000	0,000	0,000	0,000	0,000	0,000	0,000	0,210	0,120	0,000	0,000	0,000	0,000	0,000	0,000	0,000	0,000	0,000	0,000	0,000
**15**	0,000	0,000	0,000	0,000	0,000	0,000	0,000	0,000	0,000	0,000	0,194	0,000	0,000	0,000	0,000	0,000	0,000	0,000	-0,427	0,000	0,000	0,000	0,000
**16**	0,000	0,000	0,000	0,000	0,000	0,000	0,000	0,098	0,000	0,000	0,000	0,000	0,000	0,000	0,000	0,000	0,000	0,000	0,000	0,000	0,000	0,000	0,000
**17**	0,000	0,000	0,000	0,000	0,000	0,000	0,335	0,000	0,169	0,164	0,000	0,000	0,311	0,000	0,000	0,000	0,000	0,000	0,000	0,000	0,000	0,000	0,000
**18**	0,000	0,000	0,000	0,000	0,000	0,000	0,000	0,000	0,122	0,000	-0,137	0,000	0,000	0,000	0,000	0,000	0,000	0,000	0,000	0,000	0,000	0,000	0,121
**19**	0,000	0,000	0,000	0,000	0,000	0,000	0,000	0,000	0,233	0,000	0,000	0,000	0,000	0,000	-0,427	0,000	0,000	0,000	0,000	0,000	0,000	0,000	0,000
**20**	0,000	0,000	0,000	0,000	0,000	0,000	0,000	0,000	0,000	0,000	0,000	0,000	0,000	0,000	0,000	0,000	0,000	0,000	0,000	0,000	0,000	0,000	0,000
**21**	0,000	0,000	0,000	0,000	0,000	0,000	0,000	0,000	0,000	0,000	0,000	0,000	0,000	0,000	0,000	0,000	0,000	0,000	0,000	0,000	0,000	0,000	0,000
**22**	0,000	0,000	0,000	0,000	0,000	0,000	0,000	0,000	0,000	0,000	0,000	0,000	0,000	0,000	0,000	0,000	0,000	0,000	0,000	0,000	0,000	0,000	0,000
**23**	0,000	0,000	0,000	0,000	0,000	0,000	0,000	0,000	0,000	0,000	0,000	0,000	-0,193	0,000	0,000	0,000	0,000	0,121	0,000	0,000	0,000	0,000	0,000

Figure S1: Bootstrapped confidence intervals for edge weight accuracy

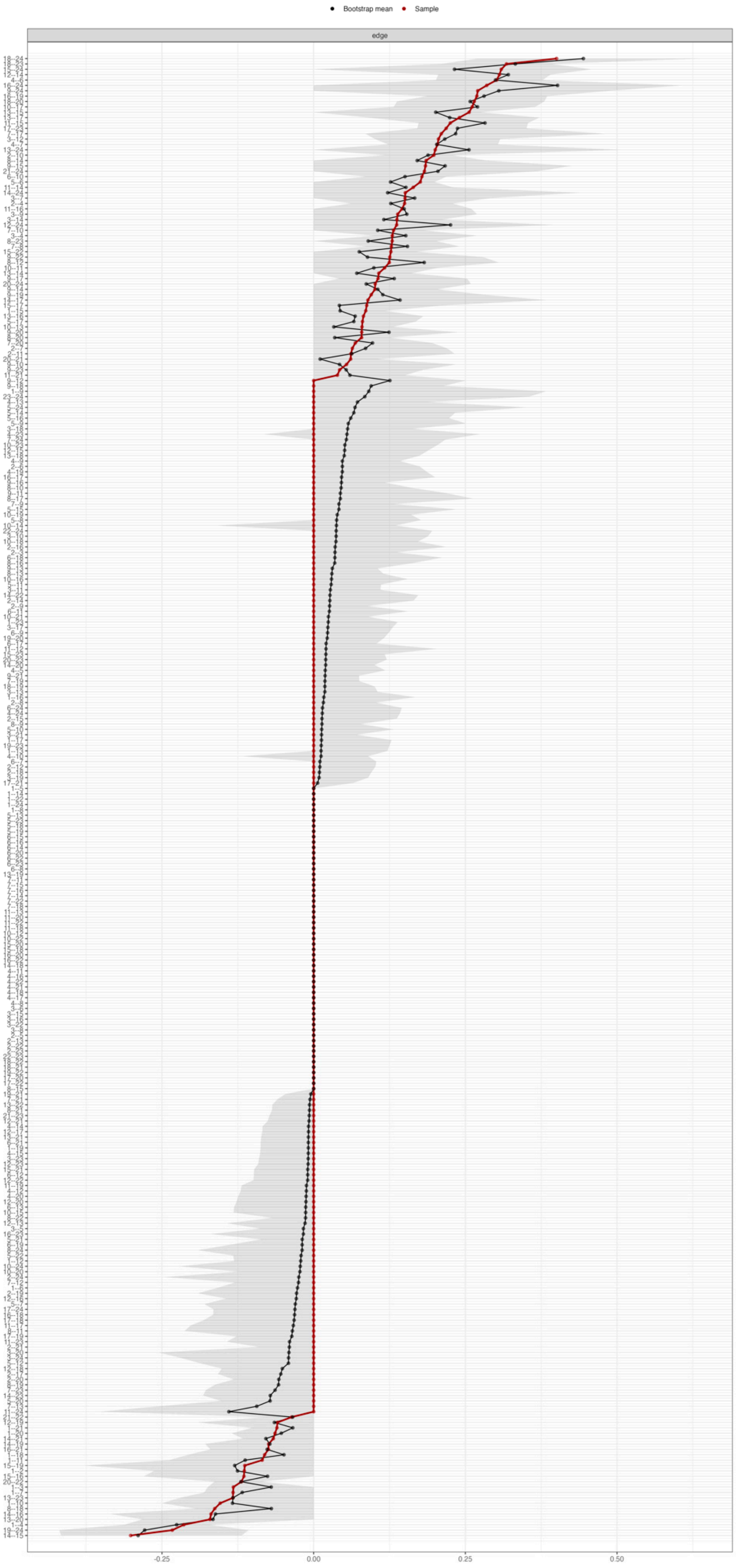

Figure S2: Bootstrapped difference test between node strengths, betweenness and closeness. Gray boxes indicate nodes that are not significantly different from one another, and black boxes indicate nodes that are significantly different from one another.

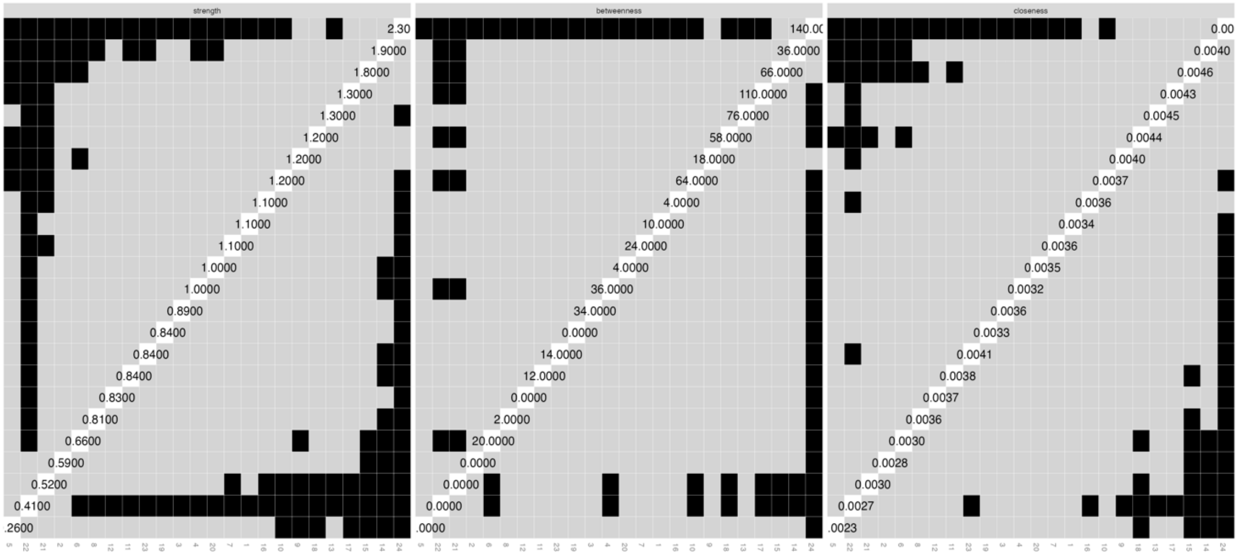

Legend of Variables

1: Perceived Stress
2: QoSR - Workplace Environment
3: QoSR - Private Environment
4: Psychological Wellbeing
5: Physical Activity
6: Physical Condition at Wake-Up (i)
7: Life Satisfaction (i)
8: Contribution of Life Aspects to Satisfaction and Wellbeing
9: Desire to Improve Quality of Relationships
10: Sense of Coherence
11: Autonomy
12: Social Resources
13: Transcendence
14: Aspirations: Personal Wellbeing and Relationships
15: Aspirations: Societal Impact and Personal Growth
16: Aspirations: Pursuit of Fame & Wealth
17: Presence of Meaning in Life
18: Age
19: Sex
20: Household Income
21: Education
22: Employment
23: Family Status
